# Supplementary figures and images for: microRNA-184 distribution and consequences on glial septate junctions and the blood-brain barrier
Source: PLoS One. 2025 Dec 4;20(12):e0328862. doi: 10.1371/journal.pone.0328862 (PMC12677480; doi:10.1371/journal.pone.0328862)

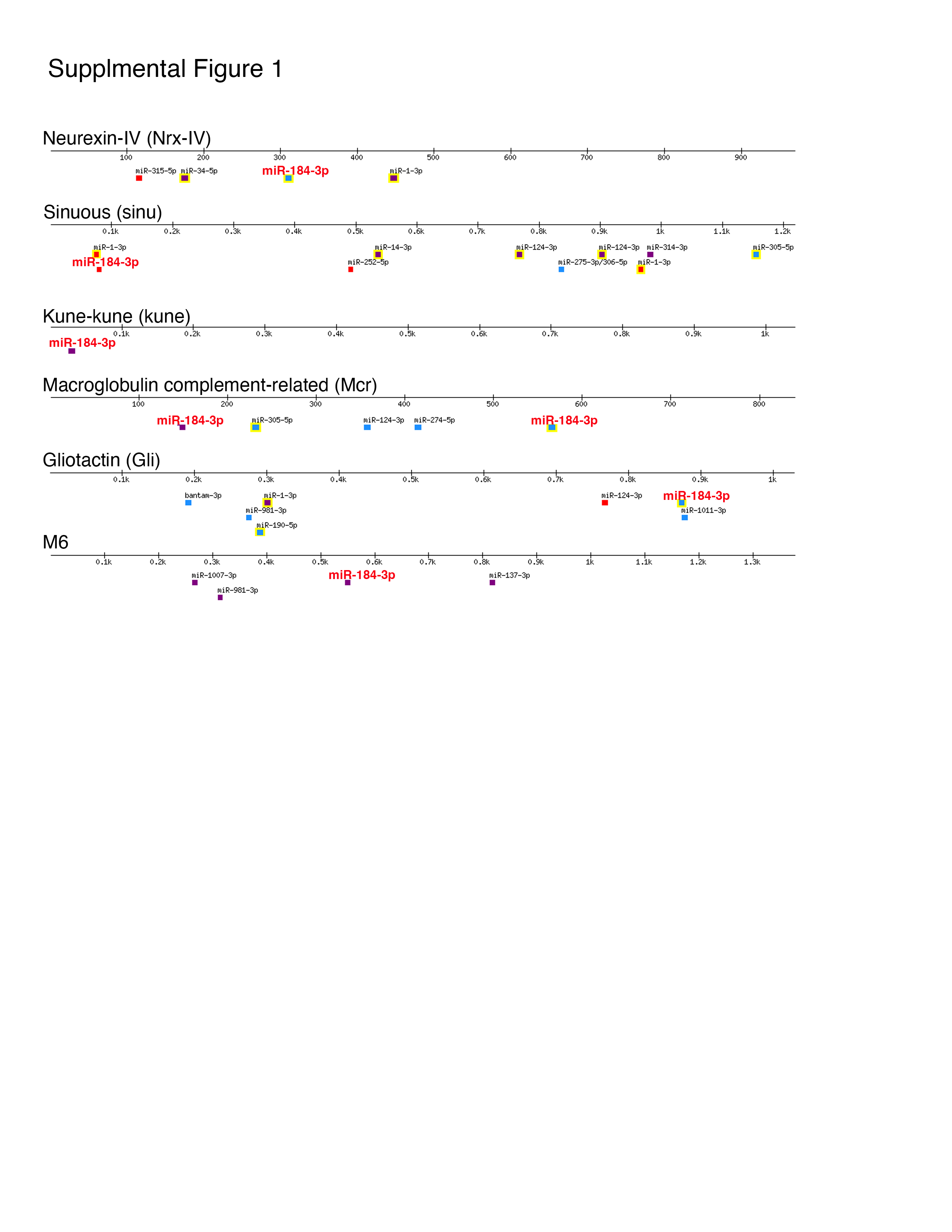

Supplement: S1 Fig — Conserved miRNA sites in the 3’ UTR of Nrx-IV, sinu, kune, Mcr, Gli and M6 predicted by target scan (www.targetscan.org/fly_72/) are shown. miR-184 target sequences are indicated in red. (TIF) [file pone.0328862.s001.tif]
