# Supplementary material for: microRNA-184 distribution and consequences on glial septate junctions and the blood-brain barrier
Source: PLoS One. 2025 Dec 4;20(12):e0328862. doi: 10.1371/journal.pone.0328862 (PMC12677480; doi:10.1371/journal.pone.0328862)
Supplement: Attachment — Submitted filename: Response to Reviewers.docx [file pone.0328862.s004.docx]

Please find below our response to the reviewers.

Thanks to the reviewers for their supportive comments and supportive suggestions. We have outlined the revisions and responses to both reviewers below.

Reviewer #1:

Minor comments.
*1. I suggest including a figure that shows the miR-184 binding sites in Nrx-IV used for generating the NLS-YFP sensor construct. It would also be of interest to indicate predicted or potential miR-184 binding sites in other core pSJ components.*

We have included a new figure as supplemental (S1 Fig) showing the predicted miRNA target sequence in Nrx-IV and other core pSJ mRNAs. The actual nucleotide sequence used is included in the materials and methods.

*2. While the manuscript focuses on larval tissues, it would strengthen the study to analyze miR-184 expression in the adult BBB to rule out expression later in development.*

This is a very good idea however the focus of this manuscript was on the larval expression and levels. This was mostly because our expertise (using the Gal4 drivers and pSJ markers) and the majority of literature is focused on the 3^rd^ instar stages thus allowing us to better interpret the effects miRNA-184 in this context.

*3. The authors report that misexpression of miR-184 using SPG-GAL4 (moody-GAL4) is lethal. However, this driver is also known to be active in other tissues such as the midgut, salivary glands, and fat body. To ensure that the observed lethality results specifically from BBB defects, the experiment should be repeated using mdr65-GAL4 (R54C07), which has more restricted expression in subperineurial glia.*

This is an excellent idea and we obtained mdr65-Gal4 line and carried out the experiment. Using this driver we observed the same degree of larval lethality observed with the SPG-Gal4 driver and have now included this data in the manuscript (lines 622-623).

*4. It would be interesting to assess whether co-expression of UAS-Nrx-IV lacking the 3′UTR (thus evading miR-184 regulation) together with miR-184 can rescue the loss of other pSJ components such as Kune and Mcr. This would strengthen the conclusion that Nrx-IV is a primary functional target of miR-184 in this context.*

We do not think that Nrx-IV is the primary functional target of miR-184 and have revised the manuscript to make this clearer (lines 826-828). That being said this is an excellent suggestion and we did try in the process of this study to do exactly this experiment. But for technical reasons we were unable to obtain a clean copy of the UAS-Nrx-IV transgene including an epitope tag.

Reviewer #2: 
Minor comments:
*- It could be interpreted that the only target of miR-148 is septate junction proteins. Whether this is the case or not, it would be important to clarify this point in the introduction.*

We have clarified in the introduction (lines 73-74) that there are many other miR-184 targets and specifically noted that only 10 are predicted pSJ mRNAs.

*- Why is the Sinu label in the brain's SPG so faint, while it is clear in the SPG of peripheral nerves? Does this suggest that the SPG of peripheral nerves has a different type of SJ?*

Yes we feel that the claudin-like proteins are likely differential expressed in the CNS versus the PNS. We have emphasized this point the discussion (lines 802-804)
